# The influence of DanceSport on the mental health of patients with chronic diseases: a systematic review

**DOI:** 10.3389/fpsyg.2026.1937157

**Published:** 2026-09-21

**Authors:** Yibei Zhang, Kang Zhu, Gulandanmu Ma

**Affiliations:** 1 School of Economics and Management, Shanghai University of Sport, Shanghai, China; 2 School of Physical Education, Shanghai University of Sport, Shanghai, China

**Keywords:** chronic diseases, DanceSport, depression, mental health, systematic review

## Abstract

**Background:**

Chronic diseases have a significant influence on patients’ mental health. DanceSport, as a form of exercise that combines physical activity, musical expression, and social interaction, may serve as an effective means of improving the physical and mental health of patients with chronic diseases; however, the current evidence regarding the influence of DanceSport on the mental health of these patients remains unclear. Clarifying this relationship will provide guidance for promoting mental health and designing exercise intervention programs for individuals with chronic diseases. Therefore, this study aims to investigate the effect of DanceSport on the mental health of patients with chronic diseases.

**Methods:**

A systematic search was conducted in the PubMed, EBSCO, and Web of Science databases for English-language literature published from the inception of each database through 7 April 2026. The literature screening process consisted of three stages: removal of duplicates, title and abstract screening, and full-text review. The entire study strictly followed the PRISMA guidelines, and the methodological quality of the included studies was assessed using the Cochrane Risk of Bias Assessment Tool.

**Results:**

This study ultimately included 21 studies involving a total of 1,087 patients with chronic diseases. The results showed that DanceSport interventions had a certain impact on mental health-related indicators in patients with chronic diseases, including depression, anxiety, wellbeing, cognitive function, emotional state, self-confidence, self-efficacy, apathy, fear, loneliness, stress, feelings of shame, and frustration. Tango and Argentine tango interventions were most widely used among patients with Parkinson’s disease; relevant studies primarily reported positive changes in outcomes such as depression, wellbeing, certain cognitive functions, emotional state, and confidence in balance. In addition, ballroom dancing, samba, waltz, and comprehensive dance interventions have also improved the mental health of patients with cancer, chronic obstructive pulmonary disease (COPD), chronic heart failure, chronic stroke, and mild cognitive impairment to varying degrees.

**Conclusion:**

DanceSport interventions may help improve the mental health of patients with chronic diseases, but their effects may vary depending on the type of disease, the style of dance, and the specific mental health outcome measures. Future research could further compare the effectiveness of different dance forms and optimize DanceSport intervention programs to enhance their value in promoting mental health among patients with chronic diseases.

**Systematic review registration:**

https://www.crd.york.ac.uk/prospero/, identifier CRD420261363754.

## 1 Introduction

According to data from the World Health Organization, approximately 41 million people die each year from chronic non-communicable diseases, accounting for about 74% of total global deaths. The prevention, control, management, and rehabilitation of chronic diseases have become major challenges facing the global public health sector (World Health Organization [WHO], 2011). Chronic diseases are typically characterized by a long course of illness, a protracted nature, and a high need for long-term management. Issues such as chronic pain, limited mobility, the burden of treatment, changes in body image, and reduced social participation not only impair patients’ physical functioning but may also trigger or exacerbate mental health problems such as anxiety, depression, stress, and loneliness, further increasing patients’ disease burden and negatively influencing their quality of life (Schrijver et al., 2022). However, current practices in chronic disease management and rehabilitation still focus primarily on physiological indicators and improvements in physical functioning, with relatively insufficient attention paid to patients’ mental health and social psychological adaptation (Hajdarevic et al., 2025). In fact, chronic diseases do not merely signify a decline in physical function; they may also alter patients’ body perception, social identity, emotional regulation abilities, and sense of self-control (Barcelos de Souza et al., 2025). Therefore, exploring non-pharmacological approaches to promote mental health among patients with chronic diseases is of great significance.

In existing studies on non-pharmacological interventions, physical activity has been widely used for the rehabilitation and mental health promotion of patients with chronic diseases (Dauwan et al., 2021; Singh et al., 2023). However, most relevant studies have focused on general physical exercise, aerobic training, resistance training, and other forms of exercise, while research on dance-based interventions has primarily centered on community dance, dance therapy, or comprehensive dance activities (Emmanouilidis et al., 2021; Koch et al., 2019). Systematic reviews of specific dance forms and their effects on mental health remain relatively limited. DanceSport, also known as International Standard Dance, is a form of physical activity that combines competitive sports with artistic expression, typically comprising two major dance styles: Latin and Ballroom (Yu et al., 2025). DanceSport integrates physical movement, musical rhythm, emotional expression, and interpersonal interaction, characterized by partner coordination, distinct rhythms, strong interactivity, and prominent artistic expression. Compared to general sports, DanceSport combines physical training, emotional experiences, and interpersonal interaction, which may be more conducive to enhancing patients’ interest in participation and adherence to interventions, while promoting psychological adaptation alongside improvements in physical function (Elmslie et al., 2025; Holmes and Hackney, 2017).

Existing research indicates that DanceSport holds certain practical value in the rehabilitation of patients with chronic diseases, at the same time, partner dance forms that have evolved from DanceSport and share similar structural characteristics—such as Argentine tango and adaptive tango—are also widely practiced, providing positive benefits for the mental health of people with chronic illnesses. However, the existing evidence on this topic remains scattered. On the one hand, previous studies have largely focused on physical-level outcomes such as balance, gait, motor ability, and physical function (Kiliç and Nalbant, 2022; Lopez et al., 2023), while systematic integration of mental health outcomes has been relatively insufficient. On the other hand, relevant studies often center on a single disease or a single dance style, with their conclusions scattered across different types of chronic diseases, different dance forms, and different mental health indicators. For example, some studies have examined the effects of tango on self-confidence and self-efficacy in patients with Parkinson’s disease (Hackney et al., 2007), while others have focused on the effects of dance forms such as the waltz on wellbeing and depressive symptoms in cancer patients (Beerenbrock et al., 2020; Schmidt et al., 2018). However, there remains a lack of research that systematically synthesizes evidence on the influence of DanceSport on the mental health of patients with chronic diseases from a holistic perspective. Based on this, this systematic review aims to systematically collect and synthesize previous relevant studies to analyze the influence of DanceSport on the mental health of patients with chronic diseases. This study will further clarify the mental health outcomes primarily addressed in existing research, compare the potential psychological benefits of different forms of DanceSport, and explore the potential mechanisms through which DanceSport improves the psychological wellbeing of patients with chronic diseases. By synthesizing the existing evidence, this paper aims to provide a theoretical basis for the application of DanceSport in chronic disease rehabilitation, mental health promotion, and non-pharmacological intervention practices, as well as to offer guidance for future research regarding intervention design, dance style selection, and the establishment of outcome measures.

## 2 Methods

This systematic review was registered in the PROSPERO Prospective Registry of Systematic Reviews (CRD420261363754) and was conducted in strict accordance with the PRISMA guidelines (Page et al., 2021).

### 2.1 Literature sources and search strategies

To systematically and comprehensively assess the influence of DanceSport on the mental health of patients with chronic diseases, this study employed a systematic literature search, retrieving articles from the following three English-language databases: PubMed, EBSCO, and Web of Science. The search time frame spanned from the inception of each database to 7 April 2026, and the search was restricted to English-language publications. The search strategies for each database covered the following search terms across three domains: (1) Medical Subject Headings (MeSH) terms for chronic diseases (“chronic disease*” OR “chronic condition*” OR “chronic illness*” OR “chronic patient*” OR “cancer*” OR “stroke” OR “coronary heart disease” OR “heart failure” OR “hypertension” OR “diabetes” OR “chronic obstructive pulmonary disease” OR “COPD” OR “Parkinson disease*” OR “cardiovascular disease*” OR “musculoskeletal disorder*” OR “obesity*”OR “multiple chronic disease*” OR “claudication”), (2) DanceSport (“sport dance*” OR “ballroom dance*” OR “modern dance*” OR “latin dance*” OR “standard dance*” OR “DanceSport*” OR “dancesport*” OR “rumba” OR “cha-cha-cha” OR “cha cha cha” OR “samba” OR “paso doble” OR “jive” OR “waltz” OR “Viennese waltz” OR “tango” OR “foxtrot” OR “quickstep”) and (3) mental health issues (“mental health” OR “mood” OR “wellbeing” OR “wellbeing” OR “wellbeing” OR “self-esteem” OR “depress*” OR “anxiety” OR “stress” OR “affect” OR “emotion*” OR “quality of life” OR “psychology*”). We also examined the reference lists of included studies (i.e., backward searching) and searched for the most recent articles cited by the included studies (i.e., forward searching) to identify other potentially eligible papers. Finally, the retrieved literature was compiled and reviewed for potential relevance to determine the final set of included studies. Subject headings, free-text terms, and field settings were appropriately adjusted according to the search rules of each database to ensure the adaptability of the search strategy across different databases.

### 2.2 Inclusion criteria (PICOS)

The inclusion criteria for this review are based on the PICOS framework, and only studies that meet the PICOS criteria were considered for inclusion.

Population (P): The study population consisted of individuals diagnosed with a chronic disease. A chronic disease is defined as a health condition requiring ongoing management or having a long-term impact on physical or psychological functioning. There were no restrictions based on gender. Studies involving healthy individuals or participants not explicitly diagnosed with a chronic disease were excluded.

Intervention (I): Interventions meeting the inclusion criteria include DanceSport or structured partner dance activities with distinct DanceSport characteristics, such as coordinated partner interaction, rhythmic movements set to music, fixed movement patterns derived from ballroom dancing or other related partner dance traditions, and recognizable techniques or elements commonly found in ballroom or partner dance practice. Eligible dance forms include tango, Argentine tango, adaptive tango, ballroom dancing, standard dances such as the waltz, Latin dances, samba, and other structured partner dance interventions that meet the operational criteria of this review.

Control (C): Eligible control conditions include routine care, no intervention, waiting-list control, conventional rehabilitation, physical therapy, other forms of physical activity, or other active control conditions. For single-group before-and-after intervention studies and qualitative studies without a formal control group, the absence of a control is not considered an exclusion criterion.

Outcomes (O): Studies must report at least one psychological or mental health-related outcome measure. Eligible outcomes include depression, anxiety, subjective or psychological wellbeing, cognitive function, emotional state, self-confidence, self-efficacy, apathy, fear of falling, loneliness, stress, shame, frustration, and other explicitly reported psychological or psychosocial outcomes. Studies reporting only physical, physiological, motor, or functional outcomes without including any psychological or mental health-related outcomes were excluded.

Study design (S): Randomized controlled trials, quasi-experimental or other non-randomized intervention studies, and qualitative studies all met the inclusion criteria. Reviews, systematic reviews, meta-analyses, study protocols, conference abstracts, editorials, commentaries, case reports, and other publications lacking original empirical data were excluded. Only full-text studies published in English in peer-reviewed journals were considered.”

### 2.3 Inclusion criteria and screening process

Inclusion criteria: (1) The study population consists of individuals diagnosed with chronic diseases; included studies must report at least one psychological or mental health-related outcome; (2) Study outcomes included mental health indicators; (3) The study examined the influence of DanceSport on mental health outcomes in patients with chronic diseases; (4) The literature was published in English in a journal; (5) The publication date ranged from the database’s inception to 7 April 2026. Exclusion criteria: (1) Study participants were not patients diagnosed with chronic diseases, or data on patients with chronic diseases could not be extracted separately from a mixed sample; (2) Interventions that did not meet the operational definition of DanceSport and related structured partner dances as defined in this paper; (3) Systematic reviews, meta-analyses, narrative reviews, research protocols, editorials, commentaries, conference abstracts, animal studies, and other non-original empirical studies; (4) Literature not in English or that has not undergone peer review. Multiple extension or follow-up reports from the same study sample may be retained but will be grouped as a single independent study to avoid double-counting participants and duplicate interpretation of study results. Studies that did not meet the inclusion criteria were excluded. Next, full-text articles from studies that appeared to meet the inclusion criteria after the initial screening were obtained for further evaluation. The full-text articles remaining after screening against the inclusion criteria were subjected to further evaluation. Any disagreements arising during the screening process were resolved through discussion among the researchers.

During the screening process, all articles retrieved from the databases were imported into EndNote V.21 for management. After removing duplicate articles, two researchers independently conducted an initial screening based on titles and abstracts to exclude irrelevant studies. For articles that appeared to meet the criteria after the preliminary screening, full texts were downloaded and reviewed in detail to determine whether they would ultimately be included in this study. During the full-text screening process, if a study was found to have incomplete information, unclear interventions, or outcome measures inconsistent with the study topic, a careful judgment was made based on the inclusion and exclusion criteria. If the two researchers disagreed on whether to include a study, they reached a consensus through discussion and negotiation; if a consensus could not be reached, a third researcher would be consulted to ensure the objectivity and consistency of the screening process.

### 2.4 Data extraction

Data extraction will be conducted independently by two researchers (YBZ and KZ) using a pre-designed data extraction form. In the event of discrepancies during the extraction process, consensus will be reached through discussion and negotiation; if disagreement persists, a third researcher will be consulted to ensure the accuracy and consistency of data extraction. Pre-determined extraction items include: publication information (title, first author’s name, year of publication); participant characteristics (sample size, gender, age, nationality, type of chronic disease); intervention details (type of dance, duration per session, frequency of intervention); and outcomes (effects on psychological problems, outcomes at various time points, occurrence of adverse events, and number of dropouts in each group). If data in the included studies are unclear or missing, the corresponding authors will be contacted via email to obtain complete data. If data remain unavailable, the analysis will be conducted based on the available data, and potential implications will be discussed.

### 2.5 Description of risk of bias assessment

To assess the methodological quality and potential risk of bias in the included studies, two researchers (YBZ and KZ) independently conducted bias risk assessments. This study used the Cochrane Risk of Bias Assessment Tool for evaluation. Cochrane is no longer described as assigning a uniform quality score to all studies, but rather as a standardized framework used to systematically identify potential sources of bias (Jørgensen et al., 2016). This tool consists of seven questions to assess the risk of bias and classifies studies as “low risk,” “unclear,” or “high risk.” The seven questions are: (1) random sequence generation; (2) allocation concealment; (3) blinding of participants and staff; (4) blinding of outcome assessment; (5) missing outcome data; (6) selective reporting; and (7) other sources of bias. Two reviewers assessed all articles included in the study. Disagreements regarding the assessment were resolved through discussion with a third reviewer (GLDMM).

## 3 Results

This paper included a total of 21 studies on the effects of DanceSport on psychological issues in patients with chronic diseases (Figure 1). Of these, 10 focused on Parkinson’s disease, seven on cancer, one on chronic heart failure, one on chronic obstructive pulmonary disease, one on cognitive impairment, and one on chronic stroke.

**FIGURE 1:**
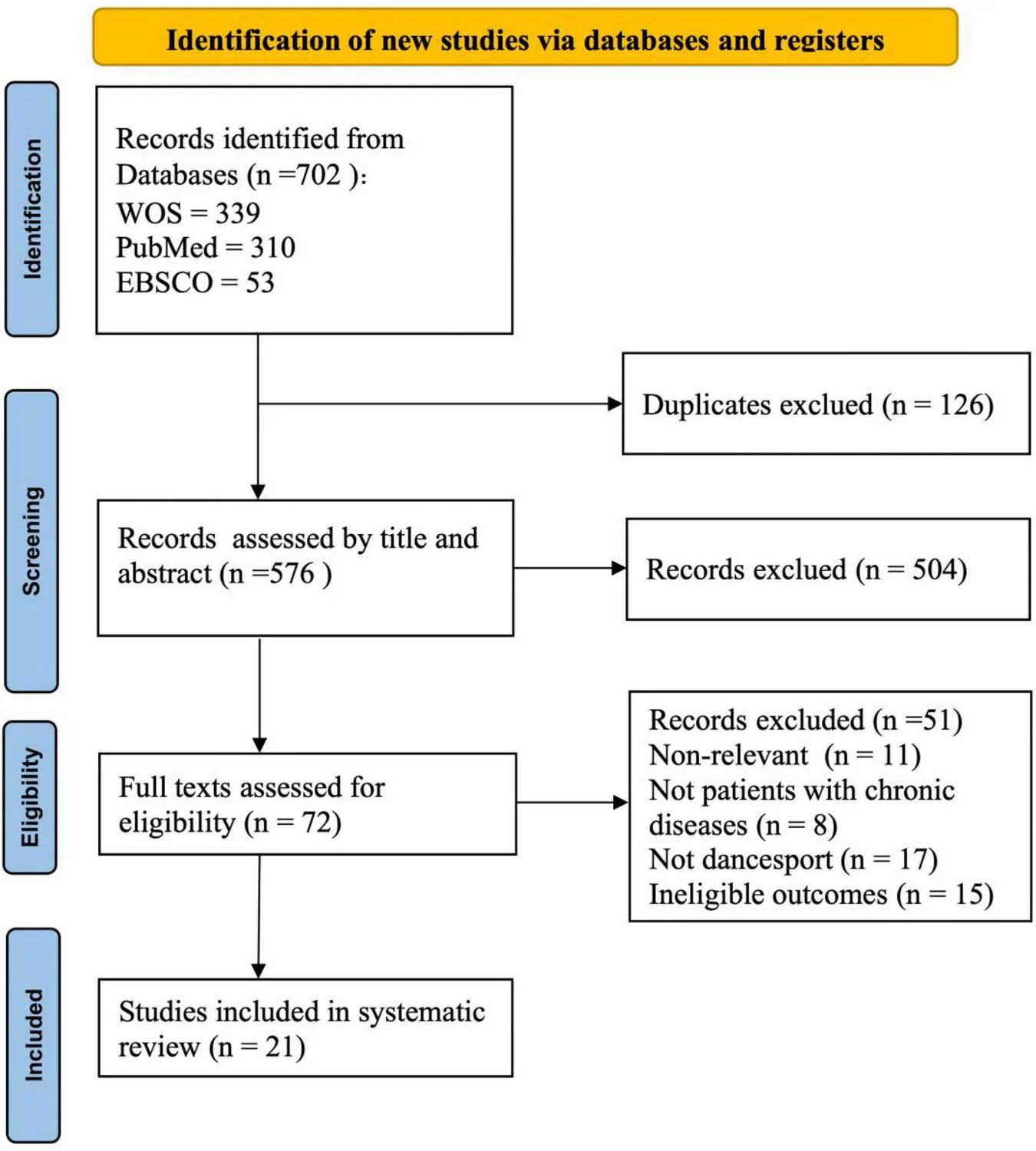
PRISMA flow diagram of study selection.

### 3.1 Participant characteristics

The total sample size across the 21 included studies was 1,087 participants, with individual study sample sizes ranging from 8 to 207 participants (Table 1). Based on the overall sample characteristics of the existing studies, participants were predominantly aged 55 and older, with the majority falling within the 56–75 age range. This indicates that DanceSport intervention studies are currently primarily directed at older adults with chronic diseases. The included studies were primarily from nine countries: Germany (*n* = 6), the United States (*n* = 4), Canada (*n* = 3), Italy (*n* = 3), Romania (*n* = 1), Brazil (*n* = 1), Switzerland (*n* = 1), Australia (*n* = 1), and the Philippines (*n* = 1).

**TABLE 1:** Characteristics of included studies.

References	Country	Age	Sample size	Duration of intervention	Target population	Intervention measures	Result variable	Research results	Psychometric instruments	Comparison groups	Adherence
Blandy et al., 2015	Australia	<75 years	6	Twice a week, 60 min each session, for 4 weeks	Parkinson	Argentine tango	Depression, quality of life	Argentine tango may alleviate depressive symptoms in people with Parkinson’s disease.	Depression (BDI); HRQoL (EQ-5D)	No comparator (single-group pre–post study)	Attendance: 89%; attrition: 17%
Koch et al., 2016	Germany	Ages 40–82	34	90 min	Parkinson	Argentine tango	Subjective wellbeing, cognitive expectations, physical self-efficacy	Tango session may improve psychological wellbeing in people with Parkinson’s disease.	HSI-24; BSE; CEQ; therapeutic factors of arts therapies scale	No control group (single-group pre–post study)	Not formally reported; one participant was unable to complete the intervention for physical reasons.
Adrian et al., 2020	Romania	Average age 60	8	Once a week, 60 min per session, for 6 weeks	Parkinson	Tango	Cognitive, depression	Tango may be associated with reduced depressive symptoms in patients with Parkinson’s disease.	UPDRS; Berg Balance Scale (BBS)	Normal routine/no physical activity	NR (not reported)
Schmidt et al., 2018	Germany	Average age 65	22	90 min	Breast cancer	Ballroom dancing, Latin dancing, standard dancing	Sense of happiness	Ballroom dance may be associated with improvements in the physical and mental health of cancer patients.	visual analogue scale (VAS, 1–10) for wellbeing	No control group	NR (not reported)
Schild et al., 2025	Germany	Ages 30–75	51	Once a week, 90 min each time	Tumor	Ballroom dancing	Physical health, mental health, wellbeing	Ballroom dancing may enhance the physical and mental health of cancer patients.	NRS (1–10) for psychological and mental wellbeing	No control group	NR (not reported)
Oei et al., 2023	Germany	Ages 18–100	158	Twice a week, 60 min each session, for 6 weeks	Breast cancer	Argentine tango	Cancer-related fatigue quality of life	Argentine tango was related to improvements in anxiety and depressive symptoms.	EORTC QLQ-C30; Cancer Fatigue Scale–German version (CFS-D)	Breast cancer survivors who did not participate in the Tango program (*n* = 108)	50 participants completed the 6-week program; follow-up completion: 100% at 6 months and 98% at 12 months
Belardinelli et al., 2008	Italy	Average age 59 ± 11	130	Three times a week, 40 minutes each session, for 8 weeks	Chronic heart failure	Waltz	Emotions	Positive changes in mood, self-confidence, and interpersonal communication were reported following waltz dancing.	Cyber MedLife Minnesota Living with Heart Failure Questionnaire (MLHFQ)	No-exercise control group (*n* = 42)	91% ± 8% attendance in the dance group
Tillmann et al., 2020	Brazil	Experimental group: 52–79 Control group: 50–81	47	Twice a week, 60 min each session, for 12 weeks	Parkinson	Samba	Cognitive function, depression, fatigue	Samba dance is helpful in alleviating the depressive symptoms of patients with Parkinson’s disease.	MMSE; PDQ-39; PDSS; BDI; FSS	No Brazilian samba intervention/usual activities; telephone contact and monthly health-care lectures	NR (not reported)
Rabini et al., 2024	Italy	Around 68 years old	24	Twice a week, 60 min each session, for 16 weeks	Parkinson	Tango	Social cognition, emotion recognition, face recognition	Tango may improve the cognitive functions and emotional recognition of patients with Parkinson’s disease.	MoCA; PAS; GDS; AES; Ekman 60 Faces Test	Group-based physiotherapy, 60 min/session, twice weekly for 4 months	NR (not reported)
Thieser et al., 2021	Germany	One third: 56–65 years old The rest: 66–75 years old	66	Once a week, 90 min each time	Cancer	Ballroom dancing	Self-efficacy, fatigue, body image	Ballroom dance was accompanied by positive changes in self-efficacy and partner intimacy among cancer patients.	BIS; BFI; ASKU	No control group	NR (not reported)
Hackney et al., 2007	United States	>40 years old	61	Twice a week, 60 min each session, for 10 weeks	Parkinson	Argentine tango	Sense of happiness	Argentine tango was linked to reductions in depression, anxiety, and stress.	PDQ-39; exit questionnaire	No-intervention control	NR (not reported)
Schad et al., 2023	Switzerland	60.5 years old	60	Once a week, 60 min per session, for 6 weeks	Breast cancer	Tango	Fatigue, cognition	Positive changes in cognitive function were reported following argentine tango among breast cancer patients.	CFS-D; EORTC QLQ-C30; PSQI	Waiting-list control/no tango intervention during the 6-week comparison period	=4 of 6 sessions required for analysis; exact attendance rate NR
Hackney et al., 2007	United States	55 years of age or older	38	Twice a week, 60 min each session, for 13 weeks	Parkinson	Tango	Balance, self-confidence, emotions, depression	Argentine tango has an impact on the confidence and emotions of patients with Parkinson’s disease.	ABC; MFES; PGCMS	Active control — conventional exercise program, 20 sessions, twice weekly, 60 min/session	NR; 20 sessions planned
Pisu et al., 2017	United States	=19 years old Average age: 57.9	62	Once a week, 45 min per session, for 10 weeks	Cancer	Ballroom dancing	Quality of life (emotional wellbeing) Mental health relationship satisfaction	Ballroom dancing has the potential to improve the quality of life for cancer survivors.	SF-36; Dyadic Trust Scale	Wait-list control; no intervention during the first 12 weeks	80% completed all 10 dance lessons and attended =1 practice party
Rios Romenets et al., 2015	Canada	Experimental group: 63.2 years Control group: 64.3 years	33	Twice a week, 60 min each session, for 12 weeks	Parkinson	Argentine tango	Happiness, emotions (depression, apathy, fatigue), cognition	Argentine tango is beneficial for the balance and cognitive functions of patients with Parkinson’s disease.	MoCA; BDI; Apathy Scale; FSS; PDQ-39	Self-directed exercise/wait-list control; usual pharmacological treatment plus exercise information	=75% attendance required; six participants failed to meet the minimum attendance requirement
Wshah et al., 2019	Canada	73.4 ± 7.6 years	20	Twice a week, 60 min each session, for 8 weeks	Chronic obstructive pulmonary disease	Including various forms of dance such as ballroom dancing and waltz.	Anxiety and depression, and health-related quality of life	Dance is related to the emotional health of patients with chronic obstructive pulmonary disease.	HADS; CRQ; ABC Scale	No control group (single-arm feasibility study)	Mean attendance rate: 78%
Dominguez et al., 2018	Philippines	>60 years old	207	Twice a week, 40 min each session, for 48 weeks	Cognitive impairment	Ballroom dancing	Cognitive performance	Ballroom dancing is beneficial for the cognitive abilities of patients with cognitive impairments.	ADAS-Cog; MoCA-P; BNT; GDS	Control group; no dance intervention reported	NR; 96 sessions planned (twice weekly for 48 weeks)
Cerulli et al., 2019	Italy	Ages 30–65	14	Twice a week, 60 min each session, for 16 weeks	Breast cancer	Ballroom dancing	Cognition, emotions, fatigue	Ballroom dancing has a positive impact on the mental health of breast cancer patients.	EORTC QLQ-C30; EORTC QLQ-FA12	No control group (single-group pre–post study)	No drop-outs reported; exact attendance rate NR
Elmslie et al., 2025	Canada	Average age: 62.4	18	10 weeks	Chronic stroke	Various forms of dance, including ballroom dancing.	Self-confidence, positive emotions, psychological support	Adaptive tango is associated with the confidence and loneliness of patients with chronic stroke.	Semi-structured face-to-face interviews	No control group (qualitative study)	All 18 interview participants completed the 10-week program; exact attendance/ adherence rate NR
Holmes and Hackney, 2017	United States	>18 years old	16	Twice a week, 90 min each session, for 12 weeks	Parkinson	Tango	Self-confidence, cognitive, perception	Adaptive tango is associated with increased self-confidence, reduced fear, and decreased stress in patients with Parkinson’s disease.	Focus groups; field observations/ notes	No control group (qualitative study)	NR (not reported)
Beerenbrock et al., 2020	Germany	Ages 38–82	12	Beginners <10 h Intermediate > 10 h	Parkinson	Tango	Anxiety, confidence, emotional experiences	Argentine tango may improve the cognitive abilities, and slow down the symptoms of depression in patients with Parkinson’s disease	Semi-structured interviews	No control group (qualitative study)	NR; participants had 10 or >10 h of tango experience

### 3.2 Characteristics of study designs

Overall, the included studies exhibited a certain degree of diversity in terms of study design and intervention methods. Regarding study design, the 21 studies comprised 6 randomized controlled trials, five pilot studies, five quasi-experimental studies, three qualitative studies, one cross-sectional study, and one single-group before-and-after study. The study participants primarily consisted of patients with chronic conditions such as Parkinson’s disease, cancer, chronic obstructive pulmonary disease, and chronic stroke. In terms of intervention methods, the types of dance employed in the included studies primarily included Samba, Waltz, Tango, Adaptive Tango, Argentine Tango and comprehensive dance forms represented by ballroom and Latin dances. There were some differences among the studies in the selection of specific dance forms, ranging from interventions focused on a single dance style to studies that integrated multiple dance forms into a single intervention program. Additionally, there were variations across studies in intervention duration, frequency of implementation, and the length of individual sessions. Overall, the duration of the interventions in the included studies varied widely, ranging from approximately 4–48 weeks, with the majority of studies lasting between 6 and 12 weeks. In terms of frequency, interventions were typically scheduled 1–3 times per week, with twice-weekly sessions being the most common; a few studies adopted a four-times-weekly schedule. One study added two group performance sessions to the standard intervention. Regarding the duration of individual sessions, most studies kept sessions between 45 and 90 min, with 60 and 90-min sessions being the most common; a few studies used 30-min training sessions.

### 3.3 Assessment of risk of bias

Potential sources of bias in the included studies were assessed using the Cochrane Risk of Bias Framework (Figures 2, 3). Overall, the studies provided relatively comprehensive reporting on incomplete outcome data, selective reporting, and other sources of bias. However, because the included studies encompassed a variety of designs—including randomized controlled trials, quasi-experimental studies, pilot studies, cross-sectional studies, single-group before-and-after studies, and qualitative studies—there were significant differences in their potential sources of bias. For randomized controlled trials, the risk of bias primarily relates to random sequence generation, allocation concealment, and blinding; for non-randomized studies and other study designs, relevant biases must be interpreted cautiously in light of their specific design characteristics. Given the heterogeneity in study designs, sample characteristics, control conditions, and measurement methods, this review does not assign a uniform methodological quality rating to all included studies; instead, the results of the risk of bias assessment are used as supplementary evidence to judge the reliability of studies and interpret the strength of the evidence. Therefore, when synthesizing and interpreting the results of this review, the impact of different study designs and their potential sources of bias on the reliability of the conclusions should be fully considered.

**FIGURE 2:**
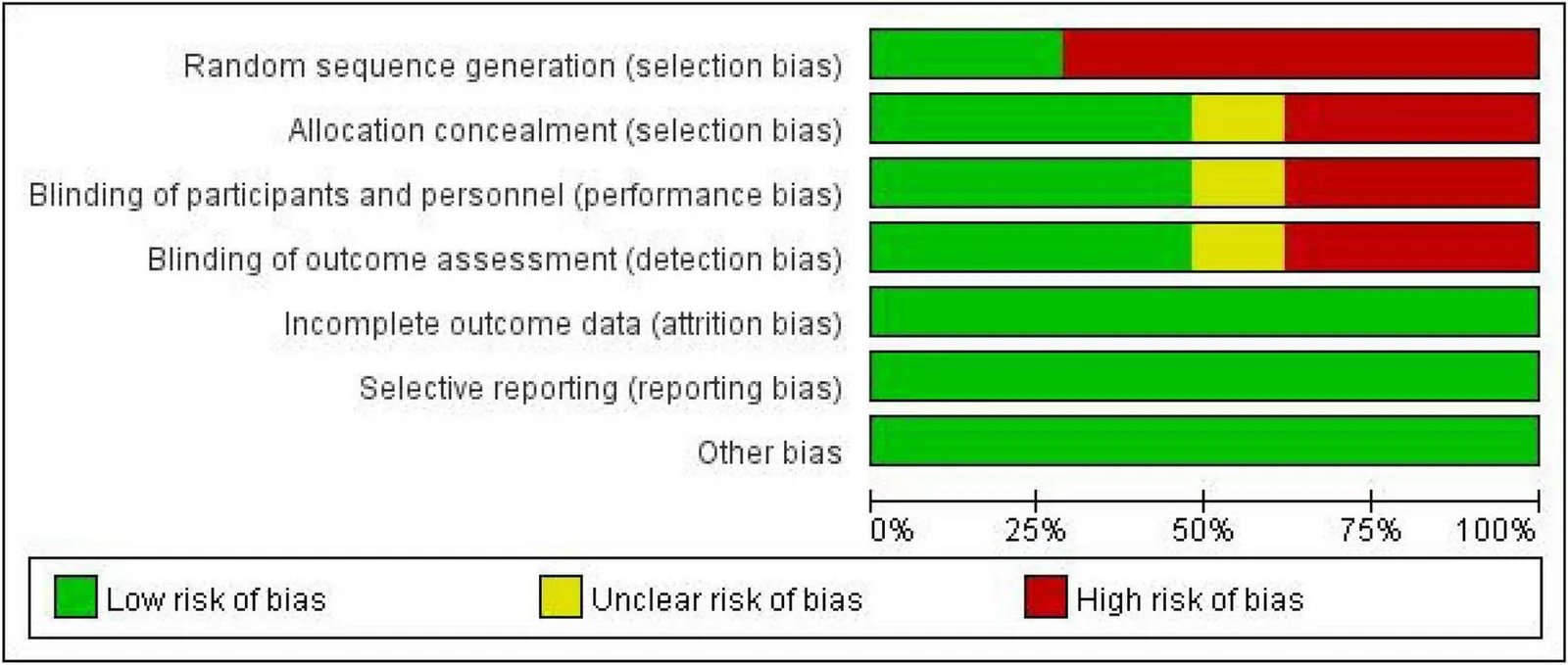
Risk of bias graph of the included studies.

**FIGURE 3:**
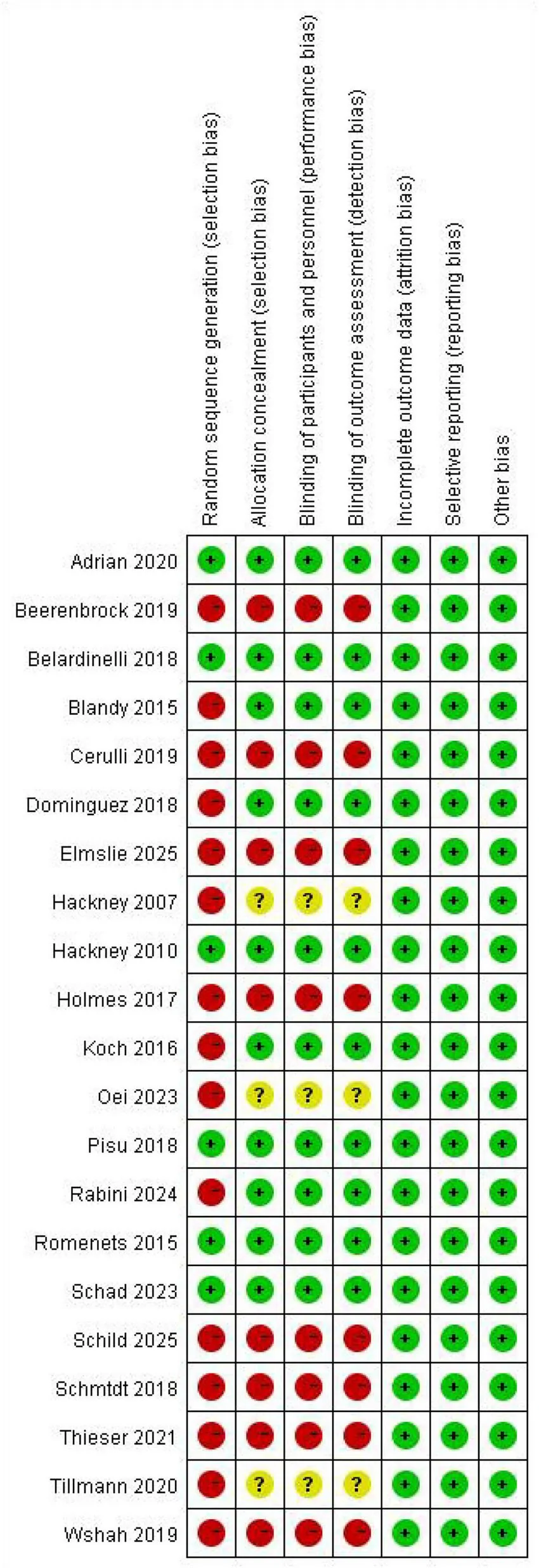
Risk of bias summary of the included studies.

### 3.4 Results of the systematic review

The outcome measures of the included studies were mental health-related, primarily focusing on depression, wellbeing, cognition, mood, anxiety, apathy, fear, stress, and loneliness. Given the similarity of the outcome measures across studies, this paper categorizes, summarizes, and synthesizes the relevant results.

#### 3.4.1 Depression

Among the included studies, a total of nine reported on the effects of DanceSport interventions on depressive symptoms (Adrian et al., 2020; Blandy et al., 2015; Dominguez et al., 2018; Hackney et al., 2007; Holmes and Hackney, 2017; Rabini et al., 2024; Rios Romenets et al., 2015; Tillmann et al., 2020; Wshah et al., 2019). The study participants primarily included patients with Parkinson’s disease, chronic obstructive pulmonary disease, and older adults with mild cognitive impairment. Among these, six studies used tango as the primary intervention (Adrian et al., 2020; Blandy et al., 2015; Hackney et al., 2007; Holmes and Hackney, 2017; Rabini et al., 2024; Rios Romenets et al., 2015), one study used Brazilian samba (Tillmann et al., 2020), and two studies employed a comprehensive dance intervention consisting of ballet, ballroom dancing, waltz, and other styles (Dominguez et al., 2018; Wshah et al., 2019). Of the nine studies mentioned above, five research studies reported on changes in depression and mood-related outcomes among patients with chronic diseases following a DanceSport intervention (Adrian et al., 2020; Blandy et al., 2015; Dominguez et al., 2018; Holmes and Hackney, 2017; Tillmann et al., 2020). In particular, the study observed that depression symptom scores among patients with Parkinson’s disease decreased following tango and Argentine tango interventions, alongside changes in mood- and mental-function-related outcomes. Following a Brazilian samba intervention, scores for non-motor symptoms—such as depression—among patients with Parkinson’s disease showed a trend toward improvement, though the associated changes were relatively limited. Additionally, depression-related outcomes improved among older adults with mild cognitive impairment following a DanceSport intervention. In addition, three studies showed that DanceSport interventions failed to significantly improve depressive symptoms in patients with Parkinson’s disease (Hackney et al., 2007; Rabini et al., 2024; Rios Romenets et al., 2015); another study indicated that a comprehensive dance intervention consisting of ballroom dance, waltz, and ballet could slightly reduce depressive symptoms in patients with chronic obstructive pulmonary disease, but the effect did not reach statistical significance (Wshah et al., 2019).

#### 3.4.2 Anxiety

Among the included studies, a total of three reported on the effects of DanceSport interventions on anxiety symptoms; the study participants primarily consisted of patients with Parkinson’s disease and chronic obstructive pulmonary disease (Beerenbrock et al., 2020; Rabini et al., 2024; Wshah et al., 2019). Of these, two studies used tango as the primary intervention method (Beerenbrock et al., 2020; Rabini et al., 2024), while one study employed a comprehensive dance intervention consisting of ballet, ballroom dancing, the waltz, and other dance forms (Wshah et al., 2019). The results showed that only one study addressed anxiety-related outcomes; the study found that some people with Parkinson’s disease reported a subjective reduction in anxiety after participating in Argentine tango (Beerenbrock et al., 2020); although the other two studies observed some relief of anxiety symptoms, neither reached statistical significance (Rabini et al., 2024; Wshah et al., 2019).

#### 3.4.3 Cognition

Among the included studies, a total of nine reported on the effects of DanceSport interventions on cognitive function (Adrian et al., 2020; Beerenbrock et al., 2020; Cerulli et al., 2019; Dominguez et al., 2018; Holmes and Hackney, 2017; Rabini et al., 2024; Rios Romenets et al., 2015; Schad et al., 2023; Tillmann et al., 2020). The study participants primarily included patients with Parkinson’s disease, breast cancer, chronic obstructive pulmonary disease, and older adults with mild cognitive impairment. Among these, tango was the most common intervention method, while other studies employed Brazilian samba, ballroom dancing, and comprehensive dance forms combining ballet, ballroom dancing, and the waltz. Overall, eight research studies reported improvements in cognitive characteristics, language function, emotional recognition, cognitive fatigue, and overall cognitive ability among patients with chronic diseases as a result of DanceSport (Adrian et al., 2020; Beerenbrock et al., 2020; Cerulli et al., 2019; Dominguez et al., 2018; Holmes and Hackney, 2017; Rabini et al., 2024; Schad et al., 2023; Tillmann et al., 2020). In particular, research on Parkinson’s disease has shown that positive changes in certain language-processing and social-cognitive measures were reported following tango interventions, such as improved ability to name actions and recognize facial emotions. At the same time, people with Parkinson’s disease also subjectively reported positive changes in their cognitive experiences, body awareness, and self-perception. Among older adults with mild cognitive impairment, interventions involving structured ballroom dancing and other dance forms, such as samba were associated with positive changes in overall cognitive function and language-naming abilities. In addition, ballroom dancing has been shown to enhance body awareness in breast cancer patients and alleviate cognitive fatigue; Comprehensive dance interventions incorporating elements of DanceSport have also been associated with positive changes in cognitive function in patients with chronic obstructive pulmonary disease (COPD). Additionally, one study indicated that while tango interventions showed a certain trend toward improving cognitive function in patients with Parkinson’s disease, this trend did not reach statistical significance (Rios Romenets et al., 2015).

#### 3.4.4 Wellbeing

Among the included studies, all five studies reported mental health outcomes related to wellbeing (Hackney and Earhart, 2009; Koch et al., 2016; Pisu et al., 2017; Schild et al., 2025; Schmidt et al., 2018). The study participants primarily included individuals with Parkinson’s disease, cancer patients, and their partners. Of these, two studies used tango as the primary intervention (Hackney and Earhart, 2009; Koch et al., 2016), while three studies used ballroom dancing as the primary intervention (Pisu et al., 2017; Schild et al., 2025; Schmidt et al., 2018). Relevant research reports positive changes in patients’ subjective wellbeing, physical wellbeing, or psychological wellbeing following DanceSport interventions. Among these, positive changes in wellbeing and self-efficacy have been reported in patients with Parkinson’s disease following tango and Argentine tango interventions. Ballroom dance interventions were primarily applied to cancer patients, particularly those with breast cancer. The study findings show that following a ballroom dance intervention, patients experienced positive changes in their subjective wellbeing as well as their physical and psychological wellbeing; similar studies have also observed positive experiences of wellbeing and psychological wellbeing among breast cancer patients and their partners.

#### 3.4.5 Mood

Among the included studies, 10 reported on the effects of DanceSport interventions on mood (Beerenbrock et al., 2020; Belardinelli et al., 2008; Cerulli et al., 2019; Elmslie et al., 2025; Hackney et al., 2007; Koch et al., 2016; Oei et al., 2023; Pisu et al., 2017; Schad et al., 2023; Wshah et al., 2019). The study participants primarily included patients with Parkinson’s disease, chronic heart failure, chronic obstructive pulmonary disease, cancer survivors and their partners, and patients with chronic stroke. The interventions primarily included tango, waltz, ballroom dancing, and comprehensive dance interventions incorporating elements of DanceSport. Most studies have reported positive changes in patients’ emotional states following DanceSport interventions. Among these, research on tango has been most prevalent among patients with Parkinson’s disease; some studies have shown that Argentine tango interventions have an impact on patients’ positive emotions, vitality, and psychological wellbeing. In these studies, participants reported positive experiences such as joy, pride, relaxation, and social interaction, while feelings of anxiety, shame, and frustration were partially alleviated; One study noted that patients with chronic stroke experienced positive psychological outcomes—such as pleasure, self-expression, and social connection—following an adaptive ballroom dance intervention; comprehensive dance interventions incorporating elements of DanceSport produced positive changes in the emotional state of patients with chronic obstructive pulmonary disease (COPD); and participation in ballroom dance can influence emotional fatigue in populations such as breast cancer patients and those with chronic heart failure. At the same time, some studies have shown that certain patients with Parkinson’ s disease may experience some negative emotional reactions during tango interventions.

#### 3.4.6 Self-confidence

Among the included studies, a total of five reported on the effects of DanceSport interventions on self-confidence or confidence in balance (Beerenbrock et al., 2020; Elmslie et al., 2025; Hackney et al., 2007; Holmes and Hackney, 2017; Wshah et al., 2019). The study participants primarily included patients with Parkinson’s disease, chronic stroke, and chronic obstructive pulmonary disease. Of these, four studies used tango as the primary intervention (Beerenbrock et al., 2020; Elmslie et al., 2025; Hackney et al., 2007; Holmes and Hackney, 2017), while one study employed a comprehensive dance intervention consisting of ballet, ballroom dancing, the waltz, and other styles (Wshah et al., 2019).

#### 3.4.7 Self-efficacy

Among the included studies, one reported on the effects of DanceSport interventions on self-efficacy (Thieser et al., 2021). The participants were cancer patients and their partners, and the primary intervention was ballroom dancing. The results showed that cancer patients’ self-efficacy improved following the ballroom dance intervention.

#### 3.4.8 Apathy

Among the included studies, two reported on the effects of DanceSport interventions on symptoms of apathy (Rabini et al., 2024; Rios Romenets et al., 2015). The participants in both studies were patients with Parkinson’s disease, and tango was the primary intervention method in both. Overall, both studies indicated that tango interventions can help improve apathy in patients with Parkinson’s disease; however, the improvements were not statistically significant in either case. Given the current limited number of relevant studies, the efficacy of DanceSport interventions in alleviating apathy symptoms in patients with Parkinson’s disease requires further validation through additional high-quality research.

#### 3.4.9 Fear

Among the included studies, one reported on the effects of DanceSport intervention on feelings of fear (Holmes and Hackney, 2017). The study results show that after participating in an adaptive tango intervention, participants experienced reduced fear and demonstrated greater physical confidence and a sense of safety during activities. However, due to the limited number of relevant studies, these findings require further support from additional empirical research.

#### 3.4.10 Feelings of loneliness

Among the included studies, one reported on the effects of a DanceSport intervention on feelings of loneliness (Holmes and Hackney, 2017). Research findings suggest that participating in adaptive tango may help alleviate feelings of loneliness, and its group-based format helped provide participants with social connection and emotional support. However, given the currently limited number of relevant studies, these findings require further validation.

#### 3.4.11 Stress

Among the included studies, one reported on the effects of a DanceSport intervention on perceived stress in patients with Parkinson’s disease, using tango as the intervention method (Blandy et al., 2015). The results showed that following the tango intervention, patients’ perceived stress was reduced, and they experienced feelings of pleasure and relaxation. Given the small number of existing studies and limited evidence, the effectiveness of DanceSport interventions in alleviating perceived stress in patients with chronic diseases requires further confirmation through high-quality research.

#### 3.4.12 Sense of shame

Among the included studies, one reported on the effects of a DanceSport intervention on the sense of shame in patients with Parkinson’s disease, using tango as the intervention method (Beerenbrock et al., 2020). The results showed that participation in Argentine tango reduced feelings of shame among people with Parkinson’s disease. However, given the limited number of relevant studies, this conclusion requires further support from additional research.

#### 3.4.13 Sense of frustration

Among the included studies, one reported on the effects of a DanceSport intervention on the sense of frustration in patients with Parkinson’s disease; the intervention involved tango (Beerenbrock et al., 2020). The results indicated that participation in Argentine tango may help alleviate feelings of frustration among people with Parkinson’s disease. However, due to the limited number of relevant studies, this conclusion requires further validation through additional research.

## 4 Discussion

This study aims to comprehensively evaluate the relationship between DanceSport-related interventions and mental health outcomes in patients with chronic diseases. Overall, existing studies have addressed multiple dimensions of mental health, including depression, anxiety, wellbeing, cognitive function, emotional state, self-confidence, self-efficacy, apathy, fear of falling, loneliness, stress, feelings of shame, and frustration; however, there are significant differences in the amount and consistency of evidence across these outcomes. Compared with previous reviews that primarily focused on a single disease, a single dance style, or physical function outcomes, this review further integrates the existing evidence from a cross-disease, cross-dance-style, and multidimensional psychological outcomes perspective, thereby expanding our understanding of the potential mental health benefits of DanceSport. In terms of the evolution of the evidence, early studies and reviews primarily focused on patients with Parkinson’s disease, with particular attention to the effects of tango and Argentine tango interventions on motor function, balance, and quality of life. In recent years, the study population has gradually expanded to include individuals with cancer, chronic obstructive pulmonary disease (COPD), chronic heart failure, and mild cognitive impairment, while mental health outcomes have broadened from depression, anxiety, and quality of life to include wellbeing, cognitive function, emotional experiences, self-efficacy, social connectedness, and fear of falling. Therefore, this review provides some support for the overall conclusion of previous studies that dance interventions may benefit the psychological and social adaptation of patients with chronic diseases. It also compares the distribution of outcomes and characteristics of evidence across existing studies in different chronic disease populations, while highlighting a significant gap in the evidence: the lack of direct comparative studies on the effects of different dance forms on mental health outcomes in patients with chronic diseases.

Depression and anxiety are common mental health issues among patients with chronic diseases and can significantly influence their quality of life. Previous studies have shown that physical exercise has a positive influence on an individual’s health and wellbeing throughout the life course (Fong Yan et al., 2024), and dance—as a comprehensive form of intervention that combines physical activity, musical stimulation, emotional expression, and social interaction—is also believed to have a positive effect on mental health (Jaldin et al., 2024). DanceSport combines the physical activity attributes of exercise with the musical, aesthetic, and interactive characteristics of dance, and therefore may offer unique advantages in alleviating symptoms of depression and anxiety. Participation in DanceSport requires physical activity at a certain intensity, which may promote the release of neurotransmitters or neuropeptides associated with emotional regulation, such as β-endorphins (Kandola et al., 2019); at the same time, the musical rhythm and aesthetic movements during dancing may activate brain regions associated with rewards processing—such as the ventral tegmental area and ventral striatum—thereby engaging the rewards system and basal ganglia circuits (Karpodini et al., 2022), triggering pleasurable experiences and promoting emotional improvement. Consequently, the rhythmic physical engagement of DanceSport, combined with musical accompaniment, partner interaction, and group dynamics, offers patients with chronic diseases a more diverse range of regulatory pathways. From a mechanistic perspective, the benefits of DanceSport for alleviating depression and anxiety involve multiple aspects. Existing research has found that regular dance training primarily increases plasma serotonin levels while decreasing plasma dopamine levels (Kiepe et al., 2012; Zhang and Ma, 2025), suggesting that dance can help restore balance to the body’s responses by regulating various neurotransmitters. At the same time, since DanceSport is performed to music, the experience of music can influence the limbic system and neurochemical circuits, promoting the generation of positive emotions (Docu Axelerad et al., 2022). Familiar music can further activate the medial prefrontal cortex and dopamine-related rewards mechanisms in the brain, which may also enhance the pleasurable experience during dance participation, thereby alleviating symptoms of depression and anxiety (Ford et al., 2016; Lee and Ko, 2023). It is worth noting that in this study, we found that tango and samba were the types of DanceSport most frequently reported to have significantly improved depression among the studies included. This may be related to their movement characteristics and emotional attributes; both tango and samba feature distinct rhythmic structures, strong bodily rhythmicity, and emotional expressiveness, which can provide a better physical experience and help increase participants’ levels of physical arousal and positive emotions (Blandy et al., 2015; Tillmann et al., 2020). This study found that DanceSport may be associated with improvements in cognition, wellbeing, and emotional state among patients with chronic diseases. This result may be related to the comprehensive intervention provided by DanceSport, which incorporates physical movement, rhythmic stimulation, emotional expression, and social interaction (Schild et al., 2025). Compared to the repetitive training of traditional sports, the process of performing DanceSport requires patients to memorize movements, adjust their physical posture, and, through partner coordination, perceive the spatial relationship and movement changes between themselves and their dance partner. This can further enhance the development of patients’ self-awareness, spatial awareness, and emotional awareness (Belardinelli et al., 2008; Dominguez et al., 2018; Rios Romenets et al., 2015). Furthermore, existing research has shown that long-term dance training may be associated with structural changes in brain regions such as the parahippocampal gyrus. This further suggests that the complex stimuli provided by dance may promote adaptive changes in the brain and enhance the activity of relevant neural networks, thereby helping to delay cognitive decline to some extent (Müller et al., 2017). In addition to potential cognitive benefits, the partner-based or group-based nature of DanceSport may further improve the psychological wellbeing of patients with chronic diseases by promoting social interaction, peer support, and emotional expression (Schmidt et al., 2018). For patients with chronic conditions, physical functional limitations, reduced social participation, and increased psychological stress are relatively common issues. The recreational and participatory nature of DanceSport provides patients with a relatively open, enjoyable, and interactive environment for engagement. Consequently, during partner or group dance sessions, participants not only gain positive emotional experiences through physical activity but also enhance their sense of belonging and social intimacy through synchronized movements and partner interaction (Lötzke et al., 2015). Existing anthropological and psychological research indicates that positive emotional experiences in synchronized dance may foster social closeness, and higher levels of social participation are typically associated with better emotional functioning, social functioning, and mental health (Durkheim and Swain, 1915). Therefore, DanceSport may not exert its effects solely through physical exercise but rather through a comprehensive pathway that promotes emotional regulation and enhances subjective wellbeing in patients with chronic diseases.

This review found that positive changes in self-confidence and self-efficacy were reported among patients with chronic diseases following DanceSport interventions, with changes in self-confidence primarily reflected in greater confidence in physical balance. Previous studies have shown that dance interventions can improve gait, balance, and flexibility in patients with neurological disorders (Hackney and Earhart, 2010; Kattenstroth et al., 2013). Since DanceSport involves intensive training in rhythm control, step transitions, and center-of-gravity adjustment, it may be particularly effective in enhancing patients’ balance abilities (Donnelly et al., 2024). Taking the tango as an example, its movements emphasize the preliminary positioning of the feet, stable support, and subsequent shifts in the center of gravity. As patients perform these movements, they must continuously perceive their body position and adjust their postural control (Beerenbrock et al., 2020). This helps enhance their perception of their own bodily control and allows them to accumulate positive balance experiences through repeated practice, thereby boosting their confidence in their balance (Adrian et al., 2020). At the same time, DanceSport movements offer a degree of flexibility and adaptability to different contexts; patients can flexibly adjust the range of motion, rhythm, and direction of their movements based on their individual conditions (Hackney et al., 2007). This not only helps reduce feelings of frustration during participation but also increases experiences of success, thereby reinforcing their positive evaluation of their own motor abilities. Furthermore, DanceSport combines aesthetic appeal with interactivity; participants must not only execute physical movements but also feel the rhythm of the music, coordinate with their dance partner, and experience the overall beauty of the dance (Fontanesi and DeSouza, 2021). Houston and Koch argue that the experience of beauty is a crucial foundation for self-efficacy, suggesting that DanceSport may further promote the development of self-efficacy through positive emotions, body identity, and social interaction (Houston, 2015; Koch, 2017). In summary, DanceSport may comprehensively enhance the self-confidence and self-efficacy of patients with chronic diseases by improving physical function, enhancing the sense of bodily control, increasing experiences of success, and providing aesthetic and interactive experiences.

This study found that a small number of studies reported potential positive changes in outcomes such as apathy, fear, loneliness, stress, shame, and frustration. As a comprehensive form of activity that integrates rhythmic movement, musical stimulation, and group interaction, DanceSport can enhance individuals’ levels of emotional arousal and positive emotional experiences, increase motivation to participate, and thereby alleviate manifestations such as apathy, diminished interest, and lack of initiative (Zhang and Ma, 2025). At the same time, dance training emphasizes footwork control, weight transfer, balance maintenance, and body coordination, which helps improve patients’ gait stability, muscle strength, and motor control abilities (Feng et al., 2022; Kumar et al., 2016). By repeatedly performing movement tasks, it also enhances their self-efficacy, thereby reducing fears of falling, loss of bodily control, or activity limitations (Veronese et al., 2017). Furthermore, DanceSport combines physical exercise with artistic expression; it can promote stress relief through aerobic metabolism and physical activity (Liu et al., 2023), and it can strengthen social connections through musical rhythm, movement expression, group practice, and peer support (Duberg et al., 2020; Liu et al., 2025), thereby alleviating feelings of loneliness and disease-related psychological burdens (Li et al., 2025). Dance also provides patients with a safe space to re-perceive, express, and accept their bodies, enabling them to develop more positive bodily experiences and body image through sustained training (Barcelos de Souza et al., 2025; Cerulli et al., 2019), thereby alleviating feelings of shame caused by illness, functional decline, or changes in appearance. Regarding feelings of frustration, the improvements in physical function, sense of motor control, and social support provided by DanceSport help patients reestablish a sense of control over their physical abilities and daily lives (Beerenbrock et al., 2020; Docu Axelerad et al., 2022). Overall, the improvement in negative psychological experiences among patients with chronic diseases through DanceSport does not stem from a single exercise effect, but rather is the result of the combined action of multiple mechanisms, including the restoration of physical function, emotional regulation, social interaction, the reconstruction of body image, and the enhancement of self-efficacy. Therefore, DanceSport can serve as a beneficial adjunctive intervention in promoting mental health and supporting rehabilitation for patients with chronic diseases.

## 5 Strengths and limitations of the study

To the best of our knowledge, this study is the first to examine the influence of DanceSport on the mental health of patients with chronic diseases. Unlike previous studies that focused on a single disease or a single dance style, this study included participants with various chronic diseases and a variety of DanceSport forms, providing a relatively comprehensive overview of the current status and potential value of DanceSport interventions in promoting mental health among patients with chronic diseases. The study results not only help deepen our understanding of the health-promoting effects of DanceSport but also provide a reference for the design, optimization, and practical application of mental health intervention programs for patients with chronic diseases. Although this study conducted a detailed analysis, the following limitations remain. First, the included studies covered a wide range of chronic diseases, and differences among patients in physical function, psychological state, and disease progression may lead to heterogeneity in intervention outcomes. Second, the DanceSport intervention programs were not entirely consistent in terms of dance style, intervention frequency, session duration, duration of the intervention, and course content; therefore, it is difficult to clearly identify the specific effects of particular dance styles and intervention dosages on different mental health outcomes. Third, this review included a variety of study designs, such as randomized controlled trials, quasi-experimental studies, pilot studies, cross-sectional studies, single-group before-and-after studies, and qualitative studies; these studies differed in terms of methodological design and strength of evidence. At the same time, the mental health outcome measures and assessment tools used in the included studies were not entirely consistent; control conditions varied widely, including no intervention, routine care, standard rehabilitation, or other active interventions; and some studies provided insufficient reporting on intervention adherence, attendance rates, or completion rates. These differences may reduce the direct comparability of study results and limit the ability to interpret the overall evidence in a unified manner. Finally, most studies focused primarily on immediate effects before and after the intervention and lacked long-term follow-up; therefore, the sustainability of the relationship between DanceSport and related structured partner dances and mental health outcomes in patients with chronic diseases remains to be further verified. Future research should further expand sample sizes, adopt more rigorous randomized controlled designs, and incorporate long-term follow-up to improve the quality of evidence and the reliability of research conclusions.

## 6 Conclusion

DanceSport interventions may be associated with positive changes in mental health outcomes among individuals with chronic diseases. Existing evidence suggests that DanceSport is associated with improvements in mental health outcomes such as depression, anxiety, stress, cognitive function, self-efficacy, and subjective wellbeing. Positive changes have also been reported in psychosocial outcomes such as apathy, shame, and loneliness, together with changes in body awareness, emotional experiences, and social connections. However, research findings still vary across different disease types, dance forms, intervention durations, and outcome measures; current evidence is insufficient to determine the relative effectiveness of various dance-based interventions or their optimal implementation conditions. Future research should further compare the mental health effects of different dance forms, intervention frequencies, durations, and exercise intensities across populations with various chronic diseases, and clarify their potential mechanisms of action, target populations, and optimal intervention conditions. This will help optimize DanceSport intervention programs and enhance their practical value in chronic disease rehabilitation and mental health promotion.

## Data Availability

The original contributions presented in this study are included in this article/supplementary material, further inquiries can be directed to the corresponding author.
